# Regulation of Reactive Oxygen Species-Mediated Damage in the Pathogenesis of Schizophrenia

**DOI:** 10.3390/brainsci10100742

**Published:** 2020-10-16

**Authors:** Samskruthi Madireddy, Sahithi Madireddy

**Affiliations:** 1 Independent Researcher, 1353 Tanaka Drive, San Jose, CA 95131, USA; 2 Massachusetts Institute of Technology, 77 Massachusetts Ave, Cambridge, MA 02139, USA; sahithim@mit.edu

**Keywords:** schizophrenia, oxidative stress, reactive oxygen species, nutrients, antioxidants, antipsychotics

## Abstract

The biochemical integrity of the brain is paramount to the function of the central nervous system, and oxidative stress is a key contributor to cerebral biochemical impairment. Oxidative stress, which occurs when an imbalance arises between the production of reactive oxygen species (ROS) and the efficacy of the antioxidant defense mechanism, is believed to play a role in the pathophysiology of various brain disorders. One such disorder, schizophrenia, not only causes lifelong disability but also induces severe emotional distress; however, because of its onset in early adolescence or adulthood and its progressive development, consuming natural antioxidant products may help regulate the pathogenesis of schizophrenia. Therefore, elucidating the functions of ROS and dietary antioxidants in the pathogenesis of schizophrenia could help formulate improved therapeutic strategies for its prevention and treatment. This review focuses specifically on the roles of ROS and oxidative damage in the pathophysiology of schizophrenia, as well as the effects of nutrition, antipsychotic use, cognitive therapies, and quality of life on patients with schizophrenia. By improving our understanding of the effects of various nutrients on schizophrenia, it may become possible to develop nutritional strategies and supplements to treat the disorder, alleviate its symptoms, and facilitate long-term recovery.

## 1. Introduction

As the most metabolically active part of the human body, the brain generates many reactive oxygen species (ROS), which can cause oxidative stress (OS) when excessively produced or inadequately removed [1,2,3]. This, in turn, can lead to neural cell damage [4,5]. Given that high oxidation activity must be balanced with antioxidant activity in the brain, it is an organ prone to oxidation-related damage [6,7,8]. Such OS is a potential factor in brain deterioration and loss of gray matter, which lead to issues with cognition and daily functioning [9,10,11,12]; furthermore, OS has been associated with a plethora of psychiatric disorders [13,14,15,16,17]. One such disorder, schizophrenia is characterized by emotional, cognitive, and behavioral disturbances, as well as inaccurate perceptions of reality and high mortality and morbidity [18,19,20,21,22]. Understanding the etiology and pathophysiology of schizophrenia is a prerequisite for developing more effective treatments. To date, the factors implicated in the development of schizophrenia include excessive free radicals and impaired antioxidant defense [5,23]. As such, a diet rich in antioxidants has been suggested as a promising strategy for slowing the progression of the disorder [24,25].

This review specifically focuses on the role of ROS-mediated oxidative damage in the pathophysiology of schizophrenia. In addition, current knowledge on treating schizophrenia with antioxidants is presented, along with information on how antioxidant levels in patients with schizophrenia may be regulated by nutritional, pharmacological, and lifestyle factors. Because schizophrenia is a progressive disorder from its onset in adolescence or early adulthood [10,26,27,28], the intake of natural antioxidants may help regulate its development [29].

## 2. Schizophrenia

Schizophrenia is a severe mental disorder characterized by frequent relapses, cognitive impairment [30,31,32], and emotional and functional disability [33,34,35]. Compared to healthy controls, patients with schizophrenia have lower total brain, gray matter, and white matter volumes and densities; on the other hand, schizophrenia patients have significantly higher third and lateral ventricle volumes [36,37]. Brain abnormalities in the amygdala, cerebellum, basal ganglia, corpus callosum, inferior parietal lobule, medial temporal lobe, superior temporal gyrus, prefrontal cortical areas, and thalamus have also been found in postmortem studies of individuals with the disorder [38,39]. The symptoms of schizophrenia are classified as positive, such as hallucinations and delusions, and negative, such as social withdrawal and flat affect [40,41,42,43,44,45]. Cognitive deficits, including the impairment of attention, memory, and executive function, are also hallmarks of schizophrenia; they present from the prodromal phase of the disorder before psychotic symptoms fully intensify [28,46,47]. Such cognitive decline can present at an early age among those with schizophrenia, eventually leading to self-care issues and impaired social and occupational function [28,48,49]. Moreover, patients with schizophrenia often report anxiety, depression, obsessive behavior, substance abuse, and suicidal ideation. Given these symptoms, schizophrenia has a high social impact [26].

## 3. Oxidative Stress

**Figure 1:**
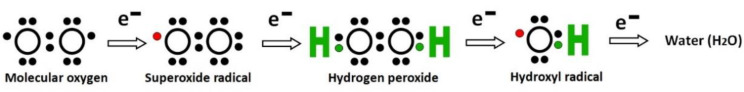
Reactive oxygen species (ROS). The continuous reduction of oxygen through adding electrons generates ROS including superoxide anion radicals (O_2_^•^), hydroxyl radicals (HO^•^), peroxyl radicals (HOO^•^), and hydrogen peroxide (H_2_O_2_). The red circle indicates an unpaired electron.

As the brain requires high levels of oxygen to function normally, it is known to be a major repository of free radicals and ROS as well as a high-risk area for neurodegeneration [15,50,51,52]. OS takes place when an imbalance arises between antioxidants and oxidants [53,54,55]. Free radicals display at least one unpaired electron and are intermediate in reducing oxygen to water [56]. Continuous reduction of oxygen causes the generation of ROS [15,57] (Figure 1). This imbalance may be attributable to a malfunctioning antioxidant system and/or high levels of ROS including superoxide anion radicals (O_2_^•^), hydroxyl radicals (HO^•^), peroxyl radicals (HOO^•^), hydrogen peroxide (H_2_O_2_), nitric oxide, and reactive nitrogen species [58,59,60,61].

**Figure 2:**
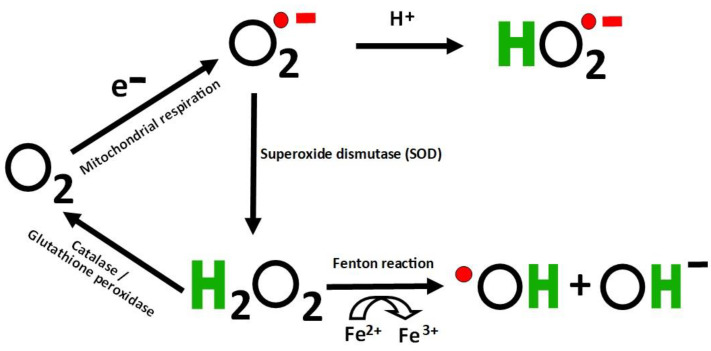
ROS generation. The superoxide is generated from oxygen in the mitochondrial respiration chain, which can be further converted into hydrogen peroxide via superoxide dismutase. The hydrogen peroxide can be transformed to hydroxyl radicals and hydroxyl anions.

O^2−^ is central to the formation of ROS; superoxide dismutases (SOD) can transform O^2−^ into the more stable H_2_O_2_ [62] (Figure 2). H_2_O_2_ can subsequently form highly reactive ·OH radicals through the Fenton reaction using Fe^2+^ as a catalyst [63]. These ·OH radicals are among the most cytotoxic and reactive ROS [64]. Conversely, H_2_O_2_ may be decomposed to water and O_2_ by catalase and peroxidases, such as glutathione peroxidase [65]. In the short-term, OS helps eradicate pathogens as part of the immune response [55]; however, severe OS caused by a major imbalance in antioxidants and oxidants causes cell damage [66,67,68]. ROS can be similarly beneficial, playing a role in modulating inflammation [2,69,70,71]; however, by modifying lipids, proteins, nucleic acids, and other molecules, excess ROS can also be damaging [61,72,73]. In particular, increased ROS may lead to lipid peroxidation, which damages cells and organelle membranes [74,75]. Furthermore, surplus ROS can also facilitate mutagenesis by causing purine oxidation, strand breaks in DNA, and cross-linking of proteins and DNA; it may also induce chromatin structure changes that can epigenetically modify gene expression [6,76,77].

## 4. Mitochondrial ROS Production

Mitochondria are responsible for cellular processes, such as energy production, cell death, and signaling [78]. They produce 90% of endogenous ROS due to leakage in electron transfer that continuously generates O_2_^−^ [79,80]. Mitochondria maintain an efficient antioxidant system to control ROS levels [63]. These levels fluctuate rapidly in mitochondria and are involved in normal cellular signaling [81]. Thus, alterations in mitochondrial redox balance due to toxins, chronic ischemia, or mutation may cause disease due to oxidative stress [81]. Neurons are particularly vulnerable to OS from ROS overproduction and deficient antioxidant responses [81]. These cells are long-lived and do not undergo mitosis. Consequently, OS that leads to mitochondrial dysfunction and eventual cell death results in loss of neuronal function [82]. Mitochondria and ROS are significant in determining how cells respond to disruption in homeostasis by stressors such as infection and metabolic changes [83]. Mitochondrial dysfunction in schizophrenia alters redox balance and produces low-grade inflammation [6]. Genetic, biochemical, and anatomical studies all provide evidence that mitochondrial dysfunction plays a role in schizophrenia [84]. Such abnormalities vary with symptoms, treatment status, and treatment response [84].

## 5. Association between Oxidative Stress and Schizophrenia

An accumulation of evidence indicates that the pathophysiology of schizophrenia is partially attributable to heightened OS [85,86,87,88,89,90]. Evidence of higher lipid peroxidation levels, changes in plasma antioxidant levels, and alterations in antioxidant enzyme activity have also been found in schizophrenia patients [91]. The oxidative imbalance in schizophrenia patients has been demonstrated through protein carbonylation, lipid peroxidation, and higher 8-hydroxydeoxyguanosine levels indicating cell death and DNA damage [10,75,92,93]. Heightened OS in those with schizophrenia can occur through disruptions to the antioxidant enzymes catalase, superoxide dismutase (SOD), glutathione, and glutathione peroxidase (GPx), [94,95,96,97,98,99,100], as well as via increased levels of the lipid peroxidation products malondialdehyde (MDA) and thiobarbituric acid reactive substances [23,85,101], and lower antioxidant levels in the cerebrospinal fluid, red blood cells, serum, and plasma [102,103]. ROS generation can also increase with schizophrenia; this has been attributed to dopamine autooxidation, mitochondrial dysfunction, and the prooxidant effects of some antipsychotic medications [104,105,106]. Mitochondrial dysfunction, which is also associated with OS, has been linked to neurodegeneration in schizophrenia [107,108,109]. Indeed, neurons are particularly vulnerable to excess ROS because of their high metabolism, plentiful fatty acids (for peroxidation), decreased antioxidant levels, reduced regenerative capabilities, and high transition metal concentrations that catalyze hydroxyl radical formation [2,15,53,110]. Given this evidence, OS could function as a biomarker for schizophrenia, indicating the pathophysiology, etiology, symptomatology, and treatment response of the disorder, and could potentially predict the progression of symptoms towards psychosis [111,112,113].

Increased oxidant activity and decreased antioxidant activity have been reported in patients with schizophrenia [107]. In a meta-analysis, researchers evaluated the evidence of OS from peripheral measures during the various clinical phases of schizophrenia [114]. A cross-sectional study comprising of 42 healthy individuals and 42 schizophrenia cases measured the total antioxidant capacity and the prooxidant antioxidant balance (which combines the prooxidant load and antioxidant capacity within one measurement) in the serum of participants; the latter value was observed to be elevated in schizophrenia patients, which indicates the prevalence of OS in the progression of schizophrenia [102]. In addition, lower antioxidant capacity indicates that schizophrenia patients may be more vulnerable to OS damage [8,102]. In another study, the relationship between SOD activity and thiobarbituric acid reactive substances in the platelets of 36 schizophrenia patients (aged 18–36) was examined in comparison to 32 healthy controls; lower antioxidative processes were observed in schizophrenia patients as well as an imbalance between prooxidants and antioxidants [115]. Furthermore, SOD activity was significantly reduced in the platelets of the patients with schizophrenia compared with the healthy controls [115]. Along with impeded antioxidant enzyme activity, numerous studies have associated decreased plasma total antioxidant status with schizophrenia [116,117,118]. For example, in a study of 50 participants with schizophrenia (aged 18–60) and 50 controls matched for age and sex, blood samples were collected to determine SOD, MDA, glutathione, and GPx levels; schizophrenia patients had significantly lower levels of SOD and GPx, but higher levels of MDA than controls, indicating increased OS [119]. Overall, evidence suggests that OS occurs in schizophrenia with an imbalance in the antioxidant defense mechanism and antioxidant enzyme impairment [120,121].

## 6. Oxidative Damage in Schizophrenia

Postmortem brains and peripheral tissues from patients with schizophrenia show evidence of OS [26], which is often the result of ROS-induced damage to macromolecules such as lipids, proteins, nucleic acids, and polysaccharides [122,123]. Studies of OS in schizophrenia have indicated the heightened oxidative damage caused by increased prooxidants and reduced antioxidants [5,124]. OS-induced damage to macromolecules ultimately affects cell damage, which is a likely factor in schizophrenia. In addition, patients with schizophrenia have been found to have weaker antioxidant defenses in their cerebrospinal fluid, peripheral blood [5,125], and postmortem brain tissue [28]. Evidence from genetic studies also suggests that these patients likely have a decreased capacity for staging a sufficient antioxidant defense response [28]. Furthermore, reports of abnormal plasma, serum, and red blood cell OS parameters also indicate that those with schizophrenia have a deficient antioxidant defense [114,126]. The inability of antioxidant defense mechanisms to combat free-radical production results in damage to cell membranes, has an adverse effect on neurotransmission [127,128], and contributes to the symptoms of schizophrenia [28,129]. Specifically, several studies have reported high nitric oxide and MDA levels, both important markers of OS, as well as reduced antioxidant glutathione levels in patients with schizophrenia [5,99,117,130]. The effects of increased cell damage and OS have also been implicated in the development of schizophrenia [131,132,133]. For example, in a study of 64 patients with schizophrenia and 80 healthy controls, in which 8-hydroxydeoxyguanosine levels, total antioxidant status, and total oxidant status were measured in plasma, OS was found to play a role in schizophrenia pathogenesis through disease damage [134]. Although reduced total antioxidant status has been observed in schizophrenia patients in several other studies, again indicating the link between the disorder and OS, similar differences have not been observed for oxidative damage of DNA [135].

## 7. Positive Effects of Nutraceuticals and Antioxidants on Schizophrenia

Given that OS-induced cell damage and the exacerbation schizophrenia symptoms can at least in part be attributed to the impairment of the antioxidant defense system [136,137], strengthening this system and scavenging free radicals by employing endogenous and exogenous antioxidants could potentially reduce the effects of OS [138,139,140]. Enzymes such as catalase, GPx, and SOD, along with vitamins E and C, are typically measured to quantify the antioxidant defense system in schizophrenia patients [115,124,130,141,142]; such antioxidants may also, therefore, have a therapeutic effect on schizophrenia [5,20,143]. Indeed, some antioxidants such as vitamins and essential polyunsaturated fatty acids have been shown to ameliorate the symptoms of schizophrenia [144,145,146]. Moreover, several studies have reported that schizophrenia can be treated with a combination of dietary supplementation, adjuvant antioxidant therapy, and antipsychotic medication [147,148].

## 8. Role of Vitamins in Schizophrenia Treatment

Adjuvant treatment including certain vitamins and minerals may have therapeutic benefits against psychiatric disorders [149,150], and there are viable biological mechanisms by which these molecules may produce their protective effects [151]. Given that quality of diet is considered a risk factor for some psychiatric disorders [152,153], it may also be possible to improve symptoms by addressing patients’ nutritional deficiencies [154]. In particular, schizophrenia patients are known to be more likely to maintain poor diets. Some preliminary evidence suggests that specific vitamin and mineral supplements could ameliorate the symptoms of schizophrenia [139].

### 8.1. Roles of Vitamin E and Vitamin C

Vitamins C and E are the most common antioxidants studied in relation to schizophrenia [124,155,156]. Schizophrenia patients have been shown to have lower plasma vitamin C and E levels compared to healthy controls [142]. Moreover, early intervention with these two vitamins, along with other antioxidants such as beta carotene, could potentially prevent oxidative damage and exacerbation of symptoms in schizophrenia, as shown by observations of lipid peroxidation and impaired antioxidant defense [157,158,159]. However, multiple studies, including 2-week to 2-year treatment periods, have failed to observe changes in Brief Psychiatric Rating Scale scores with daily doses of 600–1600 IU vitamin E [160]. Nevertheless, early studies suggest that vitamin E may help treat tardive dyskinesia, an occasional effect of long-term antipsychotic use [161]. Although a meta-analysis including 11 randomized clinical trials did not find any credible evidence for vitamin E alleviating tardive dyskinesia, some studies within the meta-analysis found that vitamin E supplementation helped prevent further deterioration of tardive dyskinesia [162]; therefore, there may be some benefit to adding vitamin E as a treatment strategy for schizophrenia [161].

Because vitamin E is lipid soluble, it has limited ability to prevent oxidative damage in the mitochondria, nucleus, and cytosolic proteins, which is where the majority of ROS are formed [163,164,165,166]. Thus, vitamin C, which is water soluble, has also been investigated as a beneficial supplement [167]. However, the adjunctive use of both vitamins in schizophrenia treatment necessitates caution since over-consumption can result in prooxidant rather than antioxidant effects [124]. The benefits of vitamin C include safeguarding neurons from OS, ensuring the proper regulation of neurotransmission, ameliorating inflation, and altering neuronal development and epigenetic function [168,169,170]. In addition to curtailing membrane phospholipid peroxidation, vitamin C can also enhance the regeneration of vitamin E [163]. Interestingly, the concentration of vitamin C in the brain is 10-fold higher than in serum; it can also be retained in the brain after crossing the blood brain barrier through GLUT1, a glucose transmitter [124]. Patients with schizophrenia have been shown to exhibit significantly reduced levels of SOD and vitamin C in comparison to healthy controls [171,172]. Therefore, supplementing with vitamin C is important: it helps maintain appropriate central nervous system (CNS) functioning, strengthens the antioxidant defense system of the brain [154], and safeguards neuronal differentiation and maturation, myelin formation, neurotransmission modulation, and catecholamine synthesis [173].

In a double-blind placebo-controlled study, 40 schizophrenia patients were split into groups receiving either vitamin C or a placebo for eight weeks, and both groups were also adjunctly treated with atypical antipsychotics [174]. While increased levels of serum MDA and reduced levels of plasma ascorbic acid were typically observed in schizophrenia patients, these levels were significantly reversed in the group treated with vitamin C relative to the placebo group. After eight weeks, there were also significantly greater improvements in the Brief Psychiatric Rating Scale (BPRS) scores of the vitamin C group compared to the placebo group [174]. Thus, oral vitamin C supplementation taken with atypical antipsychotics may lower OS, increase ascorbic acid levels, and enhance BPRS scores, signifying its potential as an adjunctive treatment for schizophrenia [174]. In another study, researchers observed that the intake of vitamin C, vitamin E, and omega-3 fatty acid supplements lowered BPRS and positive and negative syndrome scale (PANSS) scores in schizophrenia [143]. Studies involving combined treatment of vitamin E and C or vitamin C treatment alone have shown that these treatments significantly enhance BPRS scores and reduce total dyskinetic movement scores [124,174]. Both vitamins are nonenzymatic antioxidants; therefore, they likely reduce OS in schizophrenia by dismantling free-radical chain reactions [161].

#### Sources of Vitamin C and Vitamin E

Vitamin C is best obtained through certain fruits and vegetables including oranges, grapefruit, kiwis, potatoes, tomatoes, broccoli, green and red peppers, cabbage, cauliflower, strawberries, Brussels sprouts, and cantaloupe [175,176]. Dietary sources of vitamin E include nuts, such as almonds, peanuts, and hazelnuts; seeds, such as sunflower seeds; and vegetable oils, such as wheat germ oil, soybean oil, sunflower oil, and corn oil [175]. Vitamin E is also readily found in spinach, broccoli, mango, kiwi, tomato, and fortified cereals [176].

### 8.2. Role of Vitamin D

Schizophrenia patients are often deficient in vitamin D, which is known to function in neurodevelopment and neuroprotection [177,178,179,180,181,182]. The neuroprotective effects of vitamin D arise through modulation of neurotrophin production, calcium homeostasis, neuromediator synthesis, and prevention of oxidative damage [183,184,185]. A study on schizophrenia risk, which included 424 schizophrenia patients and 424 date of birth and gender matched controls, found that neonatal vitamin D levels (determined from serum 25-hydroxyvitamin (25(OH)) vitamin D3 levels in the participants’ dried blood spots gathered during the first year of their lives) influence the odds of developing schizophrenia; participants in the bottom two quintiles for vitamin D levels had a two-fold greater risk of schizophrenia compared to participants in all other quintiles [186]. In another study, in which 25(OH) vitamin D was measured in 20 recent onset schizophrenia patients and 20 matched controls, lower levels of vitamin D were linked to more severe symptoms and cognitive deficits in schizophrenia [187]. In a study of 60 patients with chronic schizophrenia, participants received either a placebo or a 50,000 IU dose of vitamin D3 once every two weeks in addition to daily consumption of probiotics (8 × 109 CFU) for 12 weeks; the combined treatment of vitamin D and probiotics had a beneficial impact not only on metabolic profiles but also on total and general PANSS scores [188].

#### Sources of Vitamin D

Few natural foods are good sources of vitamin D; however, fatty fish, such as tuna, salmon, and mackerel, as well as fish liver oils, are considered to be optimal sources [175]. Vitamin D is also available in limited quantities in egg yolks, cheese, and beef liver, primarily via vitamin D3 and 25(OH) D3 [189]. Some mushrooms offer D2, albeit in inconsistent quantities [190].

### 8.3. Role of Vitamin B

Several meta-analyses have shown that schizophrenia patients experience greater folate deficiencies than their healthy counterparts [191,192]. One study established that intake of fish fat was positively correlated with folate and vitamin B12 levels in patients with schizophrenia, suggesting that such patients can experience dietary deficits of key nutrients [193]. Further studies of schizophrenia patients have examined the potential effects of supplementation with vitamin B6 alone, folate alone, folic acid with vitamin B12, and folic acid combined with vitamins B12 and B6 [194]. An analysis of seven randomized-controlled trials that investigated vitamin B supplementation (including 297 individuals in total) found that it had a significant positive impact on total symptom scores [139]. Moreover, a 3-month supplementation with vitamin B (400 μg B12, 2 mg folic acid, and 25 mg B6) significantly lowered PANSS total scores for 42 schizophrenia patients with increased homocysteine levels [139]. In addition, the efficacy of vitamin B supplements had been shown to be significantly related to the duration of schizophrenia [145,195]: supplementation has a greater reductive effect on symptoms in the earlier stages of the disorder [139]. Overall, group B vitamin supplements, especially folate and vitamin B12, appear to improve the general symptoms of schizophrenia [196,197].

#### Sources of Vitamin B

Sources of folate include dark green leafy vegetables, nuts, fruits, peas, juices, beans, dairy, seafood, meat, eggs, grains, and poultry [175]. Specifically, folate can be found in foods such as spinach, rice, brussels sprouts, lettuce, mustard greens, green peas, wheat germ, crab, peanuts, papaya, yeast, cantaloupe, fish, ground beef, beef liver, black-eyed peas, asparagus, spaghetti, avocado, broccoli, bread, kidney beans, tomato juice, orange juice, oranges, bananas, eggs, baked beans, milk, and chicken breast [175]. Many foods are also known to be good sources of vitamin B6 including starchy vegetables, such as potatoes, non-citrus fruit, fish, fortified cereals, poultry, and organ meats such as beef liver. Natural sources of vitamin B12 include fish, milk and dairy, poultry, meat, and eggs [175,198,199]. Although plant-based diets are not adequate sources of vitamin B12, vegetarians can obtain this nutrient through fortified cereals. Vitamin B12 is also found in certain nutritional yeast products [175].

## 9. Role of Polyunsaturated Fatty Acids in Schizophrenia Treatment

The properties of docosahexaenoic acid (DHA) and omega-3 eicosapentaenoic acid (EPA) are significant for psychosis-related disorders as they are known to reduce OS by modulating mitochondria, decreasing microinflammation stress, and enhancing neurotransmission of serotonin and dopamine [191,200,201]. They can also protect against toxicity due to apoptosis and regulate gene expression of brain-derived neurotrophic factor [202]. Depletion of polyunsaturated fatty acids (PUFAs) has been linked to psychosis and cognitive deficits, and it may be associated with the heightened OS observed in schizophrenia [114,203,204]; on the other hand, similar to EPA and DHA, PUFA treatment can also prevent OS [160]. Disruption of PUFA metabolism and PUFA deficiencies in red blood cell (RBC) membranes are frequently observed in schizophrenia patients [161,205,206]. Similarly, postmortem brain studies have reported lower PUFA levels in schizophrenia patients, particularly arachidonic acid and DHA [203,207]. Seven randomized clinical trials have been conducted to compare EPA supplements against placebos in schizophrenia patients being treated with antipsychotics [207]. Among these, the positive effects of EPA on primary efficiency were reported in two studies [207]. According to one study, conducted over 12 weeks with 80 patients, EPA reduced the time until a response was observed in patients experiencing non-affective psychosis; moreover, the EPA treatment group showed a 20% reduction in antipsychotic medication use [208].

Omega-3 supplementation is known to substantially improve certain psychopathologies [209,210,211,212]. Importantly, combining omega-3 EPUFAs and antioxidants, especially during the nascent stages of illness when the human brain exhibits considerable neuroplasticity, is potentially more efficacious in improving long-term clinical outcomes [211]. In schizophrenia, omega-3 fatty acid supplements significantly alleviated symptoms in four out of seven randomized clinical trials [54,161,213]. This may be because omega-3 fatty acids contain generous amounts of EPA, which has antioxidant properties [214,215,216]. In a specific study of omega-3 supplements in schizophrenia, the nutrient significantly improved the symptoms of both schizophrenia and tardive dyskinesia [161]. Similarly, a combination of vitamin E, vitamin C, and omega-3 significantly reduced the severity of positive and negative symptoms, as well as RBC-SOD levels, in chronic schizophrenia patients over a four month period [212]. Such reduced RBC-SOD levels indicate that combining antioxidants with omega-3 PUFAs can alleviate many of the symptoms associated with OS; decreased RBC-SOD levels show that excess SOD production is no longer required to compensate for high ROS concentrations [212]. Correspondingly, in a placebo-controlled randomized clinical trial in which an *n*-3 PUFA intervention was tested over 26 weeks with 71 schizophrenia patients (aged 16–35), treatment reduced the severity of symptoms [217]. In another study, haloperidol treatment was supplemented with omega-3 fatty acids (1000 mg capsules, 180 mg EPA, 120 mg DHA; taken twice daily), vitamin E (400 IU taken twice daily), and vitamin C (1000 mg taken daily) in 17 patients with schizophrenia [212]. Over a period of four months, these patients were measured with the Scale for the Assessment of Negative Symptoms, Barnes Akathisia Rating Scale, Simpson Angus Scale, and BPRS; in all four assessments, scores in follow-up visits were significantly lower than baseline scores [212].

### Sources of PUFA

Natural sources of the PUFAs EPA and DHA are anchovies, tuna, salmon, and other fatty fish [176,218]. Alpha-linolenic acid sources include nuts, vegetable oils, leafy vegetables, flaxseeds, and flaxseed oil [219,220].

## 10. Role of Antipsychotics

Clinical trials show that antipsychotic medications significantly improve psychotic symptoms for patients with schizophrenia [221,222,223]. Atypical antipsychotics may partially normalize ROS metabolism and oxidative stress [75]. Commonly used antipsychotic drugs include aripiprazole, brexpiprazole, chlorpromazine, clozapine, lurasidone, and risperidone. At doses recommended for managing acute episodes, antipsychotic drugs can alter lipid peroxidation product (TBARS) levels in plasma [75,224].

### 10.1. Aripiprazole

Aripiprazole is an effective and well-tolerated atypical antipsychotic for patients with schizophrenia [225,226,227]. The drug acts as a dopamine D2 and serotonin 5-HT1A receptor agonist and as a serotonin 5-HT2A receptor antagonist [228,229]. Aripiprazole displays high affinity for serotonin 5-HT1A and 5-HT2A receptors and dopamine D2 and D3 receptors [223]. The drug is unlikely to cause weight gain, sedation, or other changes in metabolism [226,230,231,232]. A 52-week trial of 478 patients with schizophrenia found that intramuscular injections of aripiprazole every four weeks (fixed at either 441 mg or 882 mg) was well-tolerated, making it a suitable treatment for schizophrenia [233]. One study showed that aripiprazole did not affect levels of a plasma lipid peroxidation marker but did induce some insignificant prooxidative effects at low doses [75].

### 10.2. Brexpiprazole

Brexpiprazole is an oral atypical antipsychotic drug with clinical evidence for efficacy in the treatment of schizophrenia [234,235,236]. Like aripiprazole, the drug acts as an antagonist of serotonin 5-HT2A receptors and a partial agonist for serotonin 5-HT1A and dopamine D2 receptors [236,237,238,239,240]. Phase 3 clinical trials indicate that 2–4 mg of brexpiprazole per day is effective short-term in reducing PANSS scores and alleviating symptoms of acute schizophrenia [221,241,242]. An analysis of twelve clinical trials demonstrated that brexpiprazole was superior to placebo in improving PANSS scores in patients with schizophrenia at doses of 1 mg, 2 mg, and 4 mg over six weeks [243]. Brexpiprazole is well-tolerated and is administered daily [242,244].

### 10.3. Risperidone

Risperidone is an established treatment that alleviates both positive and negative symptoms of schizophrenia [245,246,247]. Fifty-four of 60 patients showed a decrease of 20% or more in PANSS score after two months of risperidone treatment; the remaining patients showed less reduction [245]. On average, participants gained 0.84 kg of weight during treatment [245]. Other studies found that risperidone treatment may reduce serum interleukin-6, testosterone, and estradiol levels [246,248]. Also, 30 patients with schizophrenia administered stable doses of risperidone displayed significantly reduced plasma total antioxidant capacity and increased TBARS levels compared to 30 healthy controls [249]. However, incubating control plasma with risperidone at doses commonly used in treatment produced no changes in lipid peroxidation [249].

### 10.4. Lurasidone

Lurasidone is an atypical antipsychotic with high affinity for dopamine D2 receptors, noradrenaline alpha-2C receptors, and serotonin 5-HT2A, 5-HT1A, and 5HT7 receptors [222,250,251]. A study of 2373 patients with acute schizophrenia found lurasidone was safe and effective, especially with a daily dose of 80 mg [251]. Lurasidone administered at 40 mg and 80 mg daily in adolescent patients with schizophrenia in another study found statistically and clinically significant improvement of symptoms at both doses [252]. Further, lurasidone treatment was linked to significant improvement in PANSS total scores [253]. Similarly, daily administration of 40–120 mg lurasidone for up to 24 months shows sustained improvement in PANSS total scores [254]. In general, lurasidone is well-tolerated with limited impact on weight gain and metabolism [252,253,255].

### 10.5. Clozapine

Clozapine may be a useful option for controlling symptoms in patients with first-episode schizophrenia who have not previously received treatment [256,257,258]. Clozapine is an antagonist of dopamine D2 and serotonin 5-HT2 receptors [259,260]. The drug was the first antipsychotic to show efficacy in treatment-resistant schizophrenia and shows the lowest risk of mortality [259,260,261]. A study of 36 patients indicated improved cognitive function after six months of clozapine treatment, particularly for verbal fluency and attention [262]. These effects on cognition suggest that clozapine may improve quality of life and vocational function in patients with schizophrenia [263]. Further, no significant increases were observed in plasma TBARS levels following clozapine use [264]. Finally, 100 patients with chronic schizophrenia received clozapine or risperidone as treatment [265]. Compared to risperidone, clozapine had significantly greater antioxidant effects via decreasing lipid peroxidation and increasing SOD and glutathione levels [265]. These antioxidant properties suggest that clozapine might be useful in managing negative symptoms [265,266].

### 10.6. Chlorpromazine

Chlorpromazine is commonly used globally to treat schizophrenia and is often a benchmark for evaluating alternative treatments for schizophrenia [267,268,269,270]. Data also suggest that chlorpromazine decreases lipid peroxidation by enhancing antioxidant enzyme activity [271,272].

## 11. Role of Cognitive Therapies

Nutritional and pharmacological treatment alone may be insufficient for managing all symptoms of schizophrenia, particularly negative symptoms [273]. Cognitive behavioral therapy (CBT) for psychosis is recommended alongside antipsychotics for managing schizophrenia [274,275,276]. CBT shows efficacy for lessening positive and negative symptoms over a 9-month timeframe in patients with medication-resistant schizophrenia [274,275,276]. CBT was also found effective in a study of 90 patients with schizophrenia for reducing disorganized behavior that disrupts daily routine and function [277]. CBT for patients with schizophrenia additionally decreases violent behavior, substance abuse, and suicidal ideation, while promoting physical exercise, and participation in the community [274].

Cognitive Enhancement Therapy (CET) focuses on cognitive deficits in schizophrenia, through rehabilitating cognition [278]. A study of 58 early course patients with schizophrenia over two years found CET effective [279]. CET also improves retention of employment during the early phases of schizophrenia by promoting functional recovery [279].

## 12. Role of Quality of Life

People with schizophrenia typically exhibit a poor quality of life due to their dysfunctional psychological state and lifestyle circumstances [280]. Significant issues facing people with the condition include homelessness, lack of access to medical care, poor social skills, low socioeconomic status, and unemployment [274]. Patients with schizophrenia, starting from onset, generally have low-quality diets, and nutritional deficiencies [152,281]. A factor that might partially explain this observation is that people with low socioeconomic status are eight times more likely to develop schizophrenia than people with higher socioeconomic status [282]. Moreover, nutritional deficiencies are identified as risk factors for psychiatric disorders [283]. Patients were more likely to have an irregular eating schedule, eat instant meals, and drink more coffee than controls in a study of 194 subjects [284]. People with schizophrenia also smoke four more cigarettes per day on average than controls [284]. Finally, a study of 159 patients with schizophrenia found 41% did not eat fruit daily, 51% ate meals in less than fifteen minutes, and 63% did not consume fish [285]. Inadequacies in diet and exercise associated with schizophrenia may partially be due to the disorder’s impact on socioeconomic status as many patients with schizophrenia are unable to obtain and sustain employment [286].

## 13. Implications

Pharmaceutical treatment of schizophrenia is currently limited to only a few antipsychotic medications. Notwithstanding the proven efficacy of these antipsychotics, general outcomes in schizophrenia are far from optimal. As antipsychotic drug treatment has yet to provide suitable functional recovery, there remains a need to adopt complementary approaches to treating the disorder. Therefore, adjunctive treatment options that have proven useful in schizophrenia, such as antioxidant intake, are highly desirable. The current evidence on the effects of OS in schizophrenia pathophysiology support antioxidants as a potential therapeutic strategy for this disorder.

## 14. Conclusions

Schizophrenia is unquestionably an extremely distressing brain disorder. It leads to a number of behavioral and neurodevelopmental abnormalities, including deficiencies in social functioning, perception, and processing emotions. The evidence reviewed here suggests that OS is a key component of schizophrenia’s pathophysiology, likely as a result of an imbalance between prooxidant and antioxidant molecules within cells and tissues. Evidence also suggests this imbalance is not the result of antipsychotic drug use; in some cases, antipsychotic medications have been implicated to reduce oxidative stress. Moreover, cell damage and OS are most typically present in the early stages of schizophrenia; thus, preemptive treatment with specific diets and nutritional supplements could help arrest the progression and severity of the disorder. An important consideration to a nutraceutical approach is that many schizophrenia patients have poor dietary habits, in part due to socioeconomic factors such as homelessness or unemployment. While many antioxidant treatments have been reported as efficacious in improving the psychopathology of schizophrenia, vitamins B, C, D, and E, as well as omega-3 PUFA, have perhaps emerged as the most promising complementary schizophrenia treatment strategies.
